# Undernutrition and anaemia among HAART-naïve HIV infected children in Ile-Ife, Nigeria: a case-controlled, hospital based study

**DOI:** 10.11604/pamj.2014.18.77.3746

**Published:** 2014-05-24

**Authors:** Henry Chineme Anyabolu, Ebunoluwa Aderonke Adejuyigbe, Oluwagbemiga Oyewole Adeodu

**Affiliations:** 1 Department of Paediatrics and Child Health, Obafemi Awolowo University, Ile-Ife, Nigeria

**Keywords:** Undernutrition, anaemia, HAART-naïve, HIV, Nigeria

## Abstract

**Introduction:**

Case control studies that assess the burden and factors associated with undernutrition and anaemia among HAART naïve HIV infected children in Nigeria is very sparse. This will help to formulate nutritional programs among these children.

**Methods:**

Seventy HAART naive HIV infected children aged 18 months and above were as well as seventy age and sex matched HIV negative children were recruited from August 2007 to January 2009 at Paediatric Clinic of Obafemi Awolowo University Teaching Hospital Complex, Ile-Ife, Nigeria. Their bio data, WHO clinical stage, anthropometric measurements, haematocrit, serum albumin and CD4 counts were taken with other parameters according to a study proforma.

**Results:**

The prevalence of stunting, underweight and wasting among the HIV infected subjects were 48. 6%,58. 6% and 31. 4% respectively which as significantly higher than 28. 1%, 7. 1% and 28. 1% among the HIV negative controls. 20. 1% of the HIV infected children were marasmic compared to 2. 3% of the controls. Triple anthropometric failure was found in 7. 1% of the subjects as compared to none among the controls. Anaemia is significantly more prevalent among the subjects than the controls (70. 0% vs 31. 4%; p<0. 001). The prevalence of anaemia was higher in the HIV infected subjects with undernutrition. Low socioeconomic status, hypoalbuminemia and severe immunosuppression are significantly associated with higher undernutrition prevalence.

**Conclusion:**

Several years after availability of HAART, undernutrition and anaemia remain widely prevalent among newly presenting HAART naïve HIV infected Nigerian children. Nutritional supplementation and evaluation for anaemia still need close attention in the management of these children.

## Introduction

With an estimated 75,000 new child infections through mother to child transmission in 2010, [1] Nigeria has the highest mother-to-child human immunodeficiency virus (HIV) transmission burden in the world. Many studies have shown that HIV infection worsens along with deterioration in the nutritional status of HIV infected children [2, 3]. Also, the two pathologies do interact to significantly increase mortality in these children [4, 5]. Initiating highly active antiretroviral drugs (HAART) has been shown to improve the nutritional status of HIV infected children [6, 7] but, in Nigeria, very few (less than 15%) of children in need of HAART are currently receiving them [8]. Moreover, research has shown that HAART alone is not enough to address nutritional problems in HIV infected persons [9]. Knowledge of the baseline nutritional status of HAART naïve HIV infected children will guide a nutritional plan of management that will either precede or accompany HAART. Anaemia is the most common haematologic manifestation of HIV with nutritional deficiencies, opportunistic infections and myelosuppression ranking among the major underlying causes [10]. Calis et al in a meta-analysis [11] showed that anaemia was a common complication occurring in 50-90% of children living with HIV in both resource-limited and resource-rich setting. It is also associated with poor quality of life [12]and increased mortality in HIV infected children. [10]The PEARLS Study done across 3 continents has demonstrated that the prevalence of pre-ART anaemia is different even among diverse developing countries. [13] Hence, studies that are country- specific are needed to enable policy formulation for the particular country. Studies and surveys in Nigeria on the nutritional status and burden of anaemia in children have not evaluated HIV infected children as a specific group in relation to HIV uninfected children [14–18]. Moreover, recent review on anaemia in HIV infected children confirmed that there was no case-controlled study on anaemia in HIV infected children in Africa from their literature search [10]. This study set out to determine, by a cross-sectional case-control study, the nutritional status of HAART naïve HIV infected Nigerian children presenting at an ART facility in Ile-Ife children presenting at an ART facility in Ile-Ife, South Western Nigeria. The prevalence and factors associated with anaemia were also evaluated.

## Methods

This was a hospital based cross-sectional, descriptive, and case-control study. The study was conducted at the paediatric outpatient clinic of the Ife Hospital Unit (IHU) of ObafemiAwolowo University Teaching Hospitals Complex (OAUTHC) Ile-Ife, Osun State, Nigeria. The IHU is a three hundred bedded tertiary health facility. The paediatric outpatient clinic serves as the first port of call for most of the referred, transferred and newly identified HIV infected children. HIV negative general paediatric cases are seen simultaneously as well. This was the major source of the study controls aside from other paediatric related clinics such as paediatric orthopaedic and paediatric surgery clinics. All consecutive ambulant, non-acutely ill, newly diagnosed HIV infected children who are over 18 months of age during the study period and are also not on HAART were eligible for inclusion in the study if their caregivers give consent. Eighteen months was the age cut-off as DNA-PCR was not yet available for early infant diagnosis. Seventy patients and seventy age and sex matched HIV negative control group age matched patients were recruited over the 13 months from August 2007 to January 2009 Ethical clearance was obtained from the Research and Ethical Committee of the OAUTHC and consent was obtained from parents or guardian of recruited children. All recruited patients and control were interviewed by the investigators according to the proforma to determine their age, tribe and address. The educational status were ascertained according to Oyedeji social classification method. [19, 20] The World Health Organization

Revised Clinical Staging was also used to stage each HIV infected subject. [21]Weight and height were done according to standard methods. [22] All measurements were undertaken in duplicates by one of the investigators assisted by resident doctors, after which the mean was recorded. Pre-test information and post-test counseling were offered to all the patients in compliance with WHO Guidelines on Provider- initiated Testing and counseling (PITC). [23] HIV Screening was done in the Haematology laboratory of OAUTHC by rapid testing using the parallel method according to the National Algorithm of HIV testing in use at that time [24]. The two test kits were Determine^R^ HIV-1/2 (Abbot Laboratories, Japan)and Chembio HIV 1/2 STAT-PAK^R^ (Chembio Diagnostic System Inc. New York, USA). A sample was considered positive if it was reactive to both tests kits and negative if reactive to none of the test kits. If a sample was reactive to only one of both kits it was considered indeterminate and a third test kit (Unigold) was utilised to confirm the subjects’ HIV status. Afterwards, subjects as well as controls were offered post-test counselling and then introduced to the research if they grant consent. Subjects and controls were non-fasting and the samples were collected in the morning (i. e. before 12 noon). Albumin, liver enzymes and creatinine estimation, packed cell volume, CD4 and white cell counts were determined. CD4 count was determined using automated true volumetric Cyflow machine manufactured by Partec ^®^ GMbH, Germany.

The CD4 count and CD4% were used to categorise the HIV infected subjects immunological status ranging from non-significant immunosuppression to severe immunosuppression[21]. The Wellcome classification was used to categorize cases of protein energy malnutrition in children who were less than 60 months. The World Health Organization 2007 reference standards were used to determine the z-score of weight-for-age, height-for-age and weight-for-height-for-age. Underweight, wasting and stunting were diagnosed when the weight-for-age (WA), weight-for- height (WH) and height-for-age (HA) were equal to or minus two z-scores respectively [25]. Anaemia (mild to moderate) was defined as haematocrit < 33% (equivalent to haemoglobin< 11 g/dL), and severe anaemia defined as haematocrit < 21% (equivalent to haemoglobin of < 7 g/dL) based on the WHO description of anaemia [26–29]. After all these, all the HIV positive children were subsequently placed on cotrimoxazole prophylaxis, nutritional rehabilitation and other management measures as deemed appropriate. Criteria for the initiation of HAART follow the National Paediatric ART Guideline [8] also used in other Nigerian studies [30]. This closely mirrors the WHO Paediatric ART Guideline of 2006 which was in use at the time the study was carried out [21].

Data were entered into SPSS Statistical Software Package (SPSS version 11. 0, SPSS Inc. Chicago, Ill, USA). Results were expressed as the mean ± SD. Means were compared with student's “t” test while proportions and ratios were compared using the Pearson Chi squared tests (with Yates correction for sample size less than 5 in at least one cell). Z-scores of the anthropometric measurements were calculated using the Epilnfo statistical software package (EPI INFO version 7. 0. CDC, 2011) based on WHO Child Growth Standards and WHO Reference 2007. P-values

## Results

Over thirteen months, there were a total of 2057 new cases that were seen at the clinics of the paediatric outpatient department. Two thousand and one of them accepted PITC and a hundred and nine (5. 3%) of them were HIV infected. Thirty nine of the HIV infected patients were excluded from the study mainly on grounds of acute illness at presentation (25), tuberculosis (6), haematinic supplementation usage (5) and prior HAART usage (3). Seventy of them were finally recruited for the study and complete parameters were available for all of them.

### Sociodemographic Characteristics

The study population comprised 70 HIV infected children (subjects) as well as 70 HIV negative children who served as the control. The age range of the subjects and control were the same (24 to 180 months). (Table 1) The gender ratio was similar in both subjects and control (M: F=1:1. 3; p=0. 87). Table 1 shows the distribution of the HIV infected children as well as the controls into the five socio-economic classes as described by Oyedeji [19]. There were more of the infected children in the lower socioeconomic class compared to the controls and the difference was statistically significant (p=0. 005).

**Table 1:** Demographic, clinical and laboratory profile of subjects and controls

Variables	Infected	Control
**Age Range (months**)		
**Age Group**	24 – 180	24 – 180
18-59months (n,%)	43 (61.4)	43 (61.4)
60-119months (n,%)	21 (30.0)	22 (31.4)
120-227months (n,%)	6 (8.6)	5 (7.1)
Mean Age± SD (months)	58.4± 37.8	58.01± 38.1
Gender (M vs F)		
*Social Class (n,%)	40 vs 30	39 vs 31
I	--	9 (112.9)
II	14 (20.0)	19 (27.1)
III	16 (22.9)	15 (21.4)
IV	27 (38.9)	13 (18.6)
V		
**Anthropometry Z Scores**	13 (18.6)	14 (20.0)
Median WAZ (25, 75) Male	-0.18(-0.62, 0.59)	-0.35(-0.54, 0.38)
Median WAZ (25,75) female	-0.53(-0.81, 0.36)	-0.51(-0.66, -0.15)
Median HAZ (25, 75) Male	-0.13(-0.67, 0.70)	-0.22(-0.68, 0.79)
Median HAZ (25, 75) female	-0.61(-0.88, 0.46)	-0.53(-0.83, 0.18)
Median WHZ (25, 75) Male	-1.34(-2.29, -0.21)	-0.34(-0.89, 0.09)
Median WHZ (25,75) female	-1.13(-1.87, -0.44)	-0.32(-1.11, 0.00)
#Mean ± SD Serum albumin (g/l)	3.2± 0.7	4.0± 0.5
#Mean ± SD CD4 count (cells/ml)	611.4± 444.5	1388.2± 556.5
#Mean ± SD CD4% (%)	18.8± 13.2	35.4± 4.4
#Anaemic N (%)		
**Clinical Stage (n,%)**	49 (70.0)	22 (31.4)
1	8 (11.4)	--
2	15 (21.4)	--
3	31 (44.3)	--
4		
**Immunological Stage (n,%)**	16 (22.9)	--
Not Significant Immunosuppression	4 (5.7)	--
Mild Significant Immunosuppression	15 (21.4)	--
Advanced Significant Immunosuppression	18 (25.7)	--
Severe Significant Immunosuppression	33 (47.1)	--

* Social class is by Oyedeji method (ref 19, 20)

# p value is significant (<0.05)

### WHO Clinical and Immunological Stages of the HIV infected Subjects

Table 1 shows the distribution of the HIV infected patients according to their WHO clinical and immunological stages. Forty seven (67. 1%) of them were in the late clinical stages (stages 3 and 4) while 51 (72. 9%) presented with remarkable immunosuppression (severe and advanced immunological stages).

### Nutritional Status

Table 2 shows the frequencies of stunting, wasting and underweight among the subjects as well as the controls. More of the infected children were stunted, wasted and underweight compared to the controls (p=0. 001). Twelve (8. 6%) of them had all the three (stunting, wasting and underweight) together. Table 2 also shows the Wellcome classification of the subjects and controls who are less than sixty months. Significantly increased prevalence of underweight and marasmus were seen in the subjects as compared to the controls. There was no form of kwashiorkor observed in either the subjects or the controls. The mean (+SD) of serum albumin between infected and control were also significantly different (3. 2g/l+0. 7g/l and 4. 0g/l+0. 5g/l respectively; p=0. 001) (Table 1). Moreover 40(57. 1%) of the infected subjects were hypoalbuminaemic compared to 7 (14. 9%) of the control (p=0. 001). The prevalence of undernutrition among the HIV infected subjects did not differ significantly by age group i. e. 5 years vs 5-10 years vs >10 years or gender but was higher among the lower socioeconomic classes (p=0. 006, 0. 07 and 0. 021 for underweight, wasting and stunting respectively) and the subjects with hypolbuminemia (p=0. 001, 0. 021 and 0. 007for underweight, wasting and stunting respectively). Comparing the prevalence of malnutrition across the clinical and immunological stages, there was significantly increasing prevalence of underweight (p

**Table 2:** Prevalence of malnutrition among subjects and controls by various criteria

Variables	Infected	Control	P
**Malnutrition by Anthropometry Z-core**			
Stunted (HAZ <-2)	34(48.6)	2(28.6)	0.001
Wasted (WHZ <-2)	22(31.4)	2(28.6)	0.001
Underweight (WAZ <-2)	41(58.6)	5(7.1)	0.001
**Malnutrition by Welcome Classification (Age <60months)**			
Underweight	26(60.5)	2(4.7)	0.001
Marasmus	9(20.9)	1(2.3)	0.001
Kwashiorkor	--	--	--
Marasmus Kwashiorkor	--	--	--
Normal	8(18.6)	40(93.0)	0.001
**Prevalence of multiple co-existing malnutrition (By combined anthropometry z-scores)**			
Stunting and Wasting	13(9.3)	0(0.0)	0.001
Wasting and Underweight	19(27.1)	2(2.9)	0.001
Stunting and Underweight	28(40.0)	3(4.3)	0.001
Stunting, Wasting and Underweight	12(17.1)	0(0.0)	0.001

### Anaemia

**Figure 1:**
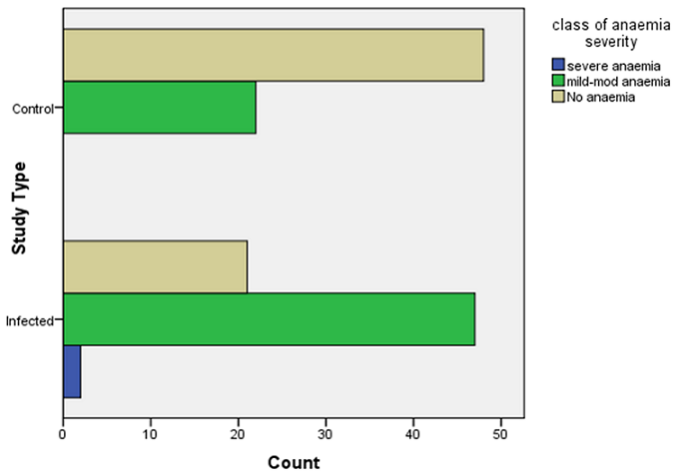
Prevalence of mild-moderate and severe anaemia in the HIV infected subjects and HIV negative controls

The prevalence of anaemia is depicted in Figure 1. It is higher in the HIV infected subjects than the controls (70. 0% versus 31. 4%; pTable 3). In relation to nutritional status, the prevalence of anaemia among the stunted in the population was 17/22 (77. 2%) compared to 54/118 (45. 6%) among the non - stunted (p=0. 015). The prevalence of anaemia among the underweight was 39/46 (84. 8%) compared to 32/94(34%) among those not underweight (p=0. 001). The prevalence of anaemia among the wasted was 20/24 (83. 3%) as compared to 51/116(43. 9%) among the non - wasted. (p=0. 001) Among the infected, the mean HAZ, WAZ and WHZ, for those with severe anaemia versus (vs) moderate anaemia vs no anaemia were -3. 8 vs -2. 1 vs 1. 5; -3. 7 vs -2. 4 vs -1. 5; -2. 5 vs -1. 6 vs -0. 7 respectively. With worsening of anaemia, there was a progressive reduction in the mean anthropometric z-scores which was significant for WAZ (p=0. 005) and WHZ (p=0. 007).

**Table 3:** Prevalence of anaemia and hypoalbuminemia, by clinical and immunological

Variable	N	Haematocrit		p-value	Serum Albumin		p-value
		Not Anaemic	Anaemic		Hypoalbuminemia	Normal albumin	
**Total**		21 (30.0%)	49(70.0%)		40(57.1)		
**Clinical stage**	70				0.016	30(42.9)	0.001
1	8	6(75.0)	2(25.0)		1(12.5)	7(87.5)	
2	15	5(33.3)	10(66.7)		6(40.0)	9(60.0)	
3	31	8(25.8)	23(74.2)		18(58.1)	13(41.9)	
4		2(12.5)	14(87.5)		15(93.8)		
**Immunological**	16			0.157†		1(6.25)	0.011
None	4	3(75.0)	1(25.0)		1(25.0)	3(75.0)	
Mild	15	6(40.0)	9(60.0)		4(26.7)	11(73.3)	
Advanced	18	4(22.2)	14(77.8)		14(77.8)		
stages among the HIV infected subjects							

## Discussion

This prevalence of malnutrition in the HIV infected subjects (58. 6%) was significantly more than in the HIV negative controls (7. 1%). This compares favourably with the prevalence of undernutrition in Cameroun (56. 4%) [29], India (55%) [27] and 56. 0% in a previous Nigerian Study [30]. It is however lower than the prevalence in Malaysia (21. 1%) [31]. The subjects in the Malaysian study being on HAART while our subjects are HAART naïve may be a contributory factor to this difference in prevalence. The prevalence of underweight, stunting and wasting in the controls were much lower than the regional average seen in the South Western Nigeria (13. 3%, 31. 2% and 7. 3% respectively) in the National Demographic Health Survey (NDHS) of 2008. [32] This could be explained by the fact that HIV screening was not done prior to the assessment of children who were involved in the survey. Hence it is possible that HIV infected children were included in the survey. Moreover, this is a hospital based study in contrad(istinction to the NDHS and majority 61. 4%) of the children who assess care at our facility are from the upper and middle socioeconomic classes. Applying the composite index of malnutrition [33], 40. 0% of the HIV infected children had both stunting and underweight, 27. 1% had both wasting and underweight while 17. 1% had triple anthropometric failure (stunting, wasting and underweight). The composite indices help to identify the children that are most in need of nutritional rehabilitation especially when resources for nutritional supplementation are insufficient. When malnutrition was assessed by the Wellcome classification among the under-fives, the prevalence of protein energy malnutrition was 35/43 (81. 4%) as compared to 3/43(7. 0%) among the controls. However, no case of kwashiorkor was seen. Kwashiorkor has been the least common form of protein energy malnutrts from Africa [34–36] and V infected children in many reports from Africa [34–37]. The most recent attempt to explain this observation is the genetically determinedheparansulfate proteoglycan (HSPG) deficiency which apparently reduces vertical transmission of HIV in infants who are predisposed to develop kwashiorkor later in life [38].

Malnutrition was significantly more in the lower socioeconomic class (IV and V) than in the upper (I and II) and middle (III) classes. This reflects similar observations in other countries [9, 39, 40] Thus, measures to address undernutrition in children with HIV should involve an improvement in the socioeconomic status of the caregivers for those efforts to be effective and have a lasting impact. As a good number of them are orphans, national and non-governmental orphan and vulnerable children (OVC) programs should place emphasis on nutritional supplementation and nutritional education of the caregivers With progressive worsening of the clinical stage and the degree of immunosuppression, the prevalence of underweight, wasting and stunting were increased. Undernutrition and HIV infection in children are interwoven, with HIV causing undernutrition, and undernutrition leading to rapid HIV disease progression [41]. In the CHAP Trial,underweight was a predictor of mortality among HIV-infected children [42]. As Sunguya et al [9] pointed out, though HAART initiation improves nutritional status, it is on its own, not sufficient to completely reverse the malnutrition present in this group of children.

The prevalence of anaemia in the subjects [70. 0%] is comparable to the values of 77. 9%, 66. 0%, 73% and 78% in Lagos [43], India [27], South Africa [44] and Malawi [45]. It is however lower than 52. 5% in another Indian study [46] where the use of HAART by the subjects may have contributed to the lower prevalence. This prevalence of anaemia observed in this study is worrisome as recent studies have identified anaemia as a significant predictor of mortality among HIV infected children [10]. Also, the prevalence of anaemia showed significant increase with progressive clinical severity and immunosuppression affecting up to 87. 5% of patients with clinical stage IV and 75. 8% of patients with severe immunosuppression. These findings are in agreement with observations in India [46], South Africa [44]. Haematocrit < 24% is a WHO Revised Clinical Stage 3 feature which is a sufficient indication to initiate HAART [21]. In this study, 37/49 (75. 5%) of those with anaemia were in either Clinical stage 3 or 4 while 39/49(79. 6%) were in the late immunologic categories (advanced and severe). Opportunistic infections, micronutrient (especially iron, folic acid and vitamin B12) deficiencies, cytokine induced myelosuppression, and HIV infection of bone marrow stroma [10] are some of the adduced reasons as all these events appear or worsen with disease progression. The high prevalence of anaemia and its equally high prevalence among the patients that present with late stages suggest that the presence and degree of anaemia should be carefully evaluated prior to HAART commencement moreso as the first line paediatric ART in most developing nations include zidovudine which is well known to induce anaemia. The case control nature of this study is its major strength. The exclusion of HIV infected children with major confounding variables such as HAART, nutritional supplementation, acute illnesses and tuberculosis is another. The lack of determination of dietary intake and food security in the homes of these children is a limitation of this study. However, the educational level and occupation of the caregivers which are the determinants of the social class in this study have a direct relationship with food security [47].

## Conclusion

This study highlights the huge burden of malnutrition and anaemia among HIV infected children in Nigeria which has remained unchanged five years after a previous report from the same centre [48]. Also, a significant proportion of these HIV infected children have multiple concurrent anthropometric failures. These are believed to be the population that are in more urgent need of supplementation in clinics with scarce resources. It has shown that social class is an important determinant of anaemia and malnutrition. Hence long term efforts to mitigate this double burden should also improve the economic status of these children. The high provider initiated counselling and testing uptake rate in this study (97. 2%) is encouraging. This shows that if access to counselling and testing is increased, more children would be diagnosed early and enrolled before the disease progresses with consequent anaemia and malnutrition. A study on the long term (5 years, for instance) impact of HAART as well as nutritional supplementation on the nutritional status and prevalence of anaemia in this centre is suggested.
